# The gut microbiota: an emerging therapeutic target for ICI-associated myocarditis

**DOI:** 10.3389/fcimb.2026.1752485

**Published:** 2026-02-19

**Authors:** Ziyin Huang, Xiaofei Mei, Yafeng Zhou

**Affiliations:** Department of Cardiology, The Fourth Affiliated Hospital of Soochow University, Suzhou Dushu Lake Hospital, Medical Center of Soochow University, Suzhou, Jiangsu, China

**Keywords:** gut microbiota, ICI, ICI-associated myocarditis, immune-related adverse events, irAEs

## Abstract

Gut microbiota and their metabolites are essential for a wide range of human physiological processes, including inflammation, immunity, and homeostasis. The intricate interplay between gut microbiota and the host immune system profoundly influences both the therapeutic response and the immune-related adverse events (irAEs) in cancer patients undergoing immune checkpoint inhibitors (ICIs) therapy. Prior evidence has established the rationale for modulating the gut microbiota to improve the incidence and prognosis of ICI-associated myocarditis. In the future, we may prevent or treat ICI-associated myocarditis by regulating the gut microbiota through methods such as microbiota transplantation, antibiotic regimens, or probiotic supplements. But there is still a considerable distance between research and clinical practice.

## Introduction

Gut microbiota and their metabolites are essential for a wide range of human physiological processes, including inflammation, immunity, and homeostasis (Valdes et al., 2018; Adak and Khan, 2019). The regulatory function of gut microbiota and their metabolites in host immunity have elicited increased scientific focus on their role in cancer-immune crosstalk and the treatment outcomes of immune checkpoint inhibitors (ICIs) (Routy et al., 2018; Lu et al., 2022; Xie and Liu, 2024). ICIs activate T lymphocyte-mediated immune responses by blocking T cell inhibitory receptors. The most widely used ICIs are monoclonal antibodies targeting programmed cell death protein 1 (PD-1) and its ligand PD-L1 (Marei et al., 2023; Kong et al., 2024; Arafat Hossain, 2024). While effectively inhibiting tumor cell proliferation, these agents may also lead to multi-organ inflammation and damage. Myocarditis has emerged as a serious immune-related adverse events (irAEs) in patients receiving ICIs treatment, in which T cells play a critical role in its pathogenesis (Mahmood et al., 2018; Moslehi et al., 2021; Munir et al., 2024). Given the established link between gut microbiota and ICIs efficacy, we hypothesize that a correlation is also likely to be existed between gut microbiota and ICI-associated myocarditis. Mechanisms of interaction between gut microbiota and ICI-associated myocarditis remain to be elucidated, and this has emerged as a critical research focus in the field of cardio-oncology. Therefore, we aim to conduct this narrative review to synthesize evidence from both preclinical and clinical studies on the association between gut microbiota and ICI-associated myocarditis. This will facilitate an exploration of the underlying molecular mechanisms and provide insights into novel intervention strategies for the treatment of this condition.

## Gut microbiota may influence the efficacy of ICIs

Considerable research has shown that gut microbiota may modify the tumor microenvironment (TME), thereby enhancing the efficacy of ICIs. Recent data have demonstrated that gut microbiota combined with ICIs acted synergistically to promote the expansion of CD8^+^, CD4^+^, and γδ T cells and reversed CD8^+^ T cell exhaustion by fostering their differentiation into memory/effector phenotypes, thereby amplifying the antitumor immune response (Cao et al., 2025). Regulatory T cells (Tregs) are a specific type of immunosuppressive T cells characterized by the expression of CD4^+^, CD25^+^, FOXP3^+^. Tregs directly suppress T cell proliferation and activation through cell-to-cell contact, and secret inhibitory cytokines with potent immunosuppressive properties, such as IL-10 and TGF-β. The proportion of Tregs was significantly decreased in these patients (Cao et al., 2025). A γδ T cell-APC(antigen-presenting cell)-CD8^+^ T cell axis was proposed to elucidate the molecular basis of the synergistic effect between gut microbiota and ICIs in immunotherapy (Cao et al., 2025). Furthermore, the gut microbiota has been reported to synthesize and transform a multitude of metabolites. These small molecules can enhance the efficacy of ICIs by modulating both local and systemic antitumor immune responses. One research published in Cell demonstrated that the tryptophan catabolite indole-3-aldehyde(I3A), released by *Lactobacillus* reuteri, locally promoted the proliferation and activation of interferon-γ-producing CD8^+^ T cells within the tumor microenvironment, thereby enhancing the efficacy of ICIs (Bender et al., 2023). Multiple studies have suggested that the gut microbiome and their metabolites may influence the efficacy of ICIs, but its association with irAEs, particularly ICI-associated myocarditis, remains insufficiently studied.

## The relationship between gut microbiota and immune-related adverse events

Recent studies have revealed that patients treated with ICIs who developed severe irAEs, including ICI-associated myocarditis had a significantly higher mean abundance of pathobionts than those who did not. Meanwhile, patients who developed severe irAEs exhibited a significantly greater decrease in the relative abundance of *Ruminococcaceae* after initiating ICIs treatment compared to those who did not develop severe irAEs (Verheijden et al., 2025). A significant correlation was also observed between *Bacteroides* and irAEs. Longitudinal analysis demonstrated a dynamic change in the relative abundance of *Bacteroides* in patients with irAEs, characterized by a decline from baseline levels during irAEs onset, followed by partial recovery upon symptom remission (Zeng et al., 2023). These findings suggested that patients who developed irAEs, including ICI-associated myocarditis do exhibit distinct alterations in their gut microbiota. These altered gut microbes may serve as potential biomarkers for predicting severe adverse events related to ICIs treatment in the future (Dora et al., 2023; Les et al., 2023).

The gut microbiota, as a critical regulator of the immune system, modulates the body’s immune response to ICIs, thereby influencing the occurrence and progression of irAEs. In the seminal preclinical study by Vétizou M et al., it was first demonstrated that *Bacteroides fragilis* within the gut microbiota played a pivotal role in modulating the development of colitis following CTLA-4 inhibitor administration (Vétizou et al., 2015). In further clinical research, investigators have also observed a correlation between the composition of the gut microbiota and the incidence of pneumonia in patients receiving PD-1 inhibitor therapy (Routy et al., 2018). The mechanisms by which the gut microbiota influences irAEs remain incompletely understood. Current mainstream hypotheses primarily encompass the following aspects. Specific gut microbiota, such as *Bacteroides fragilis*, can promote the induction and proliferation of regulatory T cells both locally in the gut and systemically. When the abundance of these beneficial microbes decreases, the immunosuppressive function may be impaired. Consequently, after ICIs release the “brakes” on the immune system, the body becomes more susceptible to excessive immune attacks against its own tissues, leading to irAEs. Conversely, other microbial species may promote the activation of effector immune cells, such as helper T cells 17 (Th17) and cytotoxic T cells. While an enhanced effector response can improve antitumor efficacy, it also increases the risk of attacking normal tissues, resulting in irAEs such as dermatitis, colitis, hepatitis, and myocarditis. Additionally, gut microbiota continuously stimulates innate immune cells via pattern recognition receptors, influencing their cytokine secretion profiles and thereby modulating the development of irAEs (McCulloch et al., 2022; Hu et al., 2024; Gao et al., 2025). Molecular mimicry and cross-reactivity also play a critical role in this process. Certain protein epitopes derived from gut commensals or pathogens share high structural similarity with epitopes of human tissues, such as those in the heart, skin, and muscles. Under ICI treatment, T cells activated against these microbial antigens may mistakenly recognize and attack human tissues with similar epitopes due to molecular mimicry, leading to organ-specific autoimmune damage. A notable example is ICI-associated myocarditis (Gil-Cruz et al., 2019).

## Mechanisms by which the gut microbiota influence ICI-associated myocarditis

Emerging evidence suggest that the gut microbiota modulates ICI-associated myocarditis through multifaceted biological pathways, though these mechanisms remain incompletely elucidated. Based on studies of other irAEs and autoimmune cardiomyopathies, we infer that the mechanisms underlying ICI-associated myocarditis primarily involve the following aspects. First, the alterations in gut microbiota may lead to the release of mimic peptides which can trigger autoimmune response against the heart in patients receiving ICIs. Aberrantly activated lymphocytes targeting self-antigens in the myocardium are the underlying cause of myocarditis (Gil-Cruz et al., 2019). Second, the gut microbiota may also amplify ICI-associated myocarditis by suppressing the anti-inflammatory function. Gut microbiota dysbiosis potentiate the decline in Tregs caused by PD-1 inhibitors, leading to an increased incidence of ICI-associated myocarditis (Cao et al., 2025). Furthermore, the gut microbiota can activate systemic pro-inflammatory cytokine networks (Du et al., 2025). For example, chemokines such as CXCL9 and CXCL10 recruit more CD8^+^ T cells, Th1 cells, and monocytes to the heart, exacerbating myocardial inflammatory infiltration. TNF-α and IFN-γ can directly induce cardiomyocyte apoptosis, while TGF-β promote the activation of cardiac fibroblasts, leading to myocardial fibrosis and impaired contractile function (Kang and Izumo, 2003; Wei et al., 2021). However, these proposed mechanisms remain investigational and warrant further validation through rigorously designed studies.

## The therapeutic application of antibiotics for ICI-induced myocarditis continues to be contentious in clinical practice

Recent studies have revealed that gut microbiota were associated with the markers of myocardial injury and participated in the pathogenesis of ICI-associated myocarditis. Cao et al. evaluated important immune cells, cytokine levels and the composition of gut microbiota under different interventions. Correlation analysis revealed that *Flexispira* abundance in PD-1 antibody-treated mice exhibited significantly positive associations with cardiac injury markers (CD4^+^ T-lymphocytes, CD8^+^ T-lymphocytes and Th17 cells), while demonstrating inverse correlations with cardioprotective factors (serum IL-10, CD25 mRNA, FOXP3 mRNA, IL-10 mRNA, FOXP3 mRNA, IL-10 mRNA, and TGF-β mRNA) (Cao et al., 2025). Meanwhile, *Ruminococcaceae* showed a positive correlation with all cardiac injury factors except Th17 cells, and a negative correlation with all cardioprotective factors, including Tregs. Furthermore, the researchers performed fecal microbiota removal in mice using a cocktail containing 0.5 g/L Vancomycin, 0.5 g/L Neomycin Sulfate, and 0.5 g/L Primaxin. Fecal microbial removal experiments demonstrated that depleting gut microbiota associated with PD-1 inhibitor-induced myocarditis through antibiotic treatment reduced inflammatory cell infiltration and diminished myocardial injury area (Cao et al., 2025). Another research investigating the relationship between gut microbiota and myocarditis was published recently. It has revealed that patients with myocarditis exhibited significantly elevated *Bacteroides*-specific CD4^+^ T cell and B cell responses. This indicated that mimic peptides derived from commensal bacteria could trigger the development of inflammatory cardiomyopathy (Gil-Cruz et al., 2019). Notably, treatment with a broad-spectrum antibiotic combination comprising sulphadoxine, trimethoprim and metronidazole successfully blocked the progression to heart failure in the mouse model, suggesting these findings hold significant translational value. Targeting the gut microbiota of patients undergoing ICIs treatment through antibiotics may prevent the onset of ICI-associated myocarditis and its potential lethal sequelae. Building upon current findings, targeted manipulation of gut microbiota through precision antibiotic regimens represents a promising frontier in mitigating cardiotoxicity induced by ICIs. However, the therapeutic application of antibiotics for managing ICI-associated myocarditis is currently supported only by preclinical evidence from animal studies, with clinical validation still pending.

At the same time, the use of antibiotics to mitigate irAEs may also compromise the efficacy of ICIs therapy. Antibiotics may facilitate the overgrowth of opportunistic pathogens, leading to the production of immunosuppressive substances or promoting the expansion of regulatory T cells, thereby suppressing the function of effector T cells. Multiple retrospective studies have found that the use of broad-spectrum antibiotics, particularly penicillin-class drugs, during ICIs treatment was associated with shortened patient survival and a significantly increased risk of tumor progression (Xu et al., 2020; Gambichler et al., 2025). Therefore, the appropriate use of antibiotics for treating and preventing ICI-associated myocarditis remains controversial. Selecting the right type and dosage of antibiotics to mitigate ICI-associated myocarditis without compromising the efficacy of ICIs therapy represents a key focus for future research.

## The regulatory effects of other commonly used drugs on gut microbiota in ICI-associated myocarditis

Clinically, patients receiving ICIs therapy may concurrently be prescribed proton pump inhibitors or statins. These medications can also modulate the gut microbiota, thereby potentially influencing the onset and progression of irAEs, including ICI-associated myocarditis. Long-term use of proton pump inhibitors is associated with reduced gut microbiota diversity and alterations in the abundance of specific microbial taxa. This dysbiosis may disrupt immune tolerance, theoretically increasing the risk of various irAEs, including myocarditis (Rassy et al., 2022; Shatila et al., 2025). Statins can suppress the overactivation of reactive T cells and mitigate systemic inflammatory responses, thereby potentially ameliorating the pathological progression of ICI-associated myocarditis (Balanescu et al., 2020). Therefore, for patients concurrently using proton pump inhibitors, statins, or similar medications, the influence of gut microbiota on ICI-associated myocarditis may differ from that in patients not taking these drugs. This highlights the need for more detailed and comprehensive future studies to clarify these potential interactions.

## Discussion

ICIs represent a class of revolutionary anticancer agents that activate the body’s anti-tumor immunity by blocking T cell inhibitory receptors (Isaacs et al., 2021; Herisson et al., 2024). However, ICIs may lead to the overactivation of immune system, resulting in normal organ damage and a series of irAEs. Among these, ICI-associated myocarditis, although relatively uncommon in incidence, is a highly fatal complication with a mortality rate as high as 25%-50% (Patel et al., 2021). It has become a major cardiovascular safety concern for cancer patients undergoing ICIs therapy. Emerging evidence reveals that gut microbiota plays a pivotal role in cardiovascular disease development through the “gut-heart axis” (Tang et al., 2017; Witkowski et al., 2020). The intricate interplay between gut microbiota and the host immune system profoundly influences both the therapeutic response and the incidence of irAEs in cancer patients undergoing ICIs therapy.

As is widely recognized, the gut microbiota modulates the efficacy of ICIs by promoting the expansion of CD8^+^, CD4^+^, and γδ T cells, while suppressing Tregs. Alterations in the gut microbiota also influence the onset and progression of irAEs, including ICI-associated myocarditis. The mechanisms by which the gut microbiota induces ICI-associated myocarditis remain incompletely elucidated [12.23]. The release of mimic peptides, which drives an autoimmune response targeting the heart, serves as the central mechanism by which gut microbiota impacts the onset of ICI-related myocarditis. Furthermore, the imbalance in gut microbiota can suppress immunosuppressive Tregs. Consequently, this results in systemic immune overactivation, which in turn influences the onset and progression of ICI-associated myocarditis. However, these mechanisms are still under investigation and require further experimental validation.

Changes in the gut microbiota, particularly the composition of specific bacterial species, affect the development of ICI-associated myocarditis. Therefore, researchers hypothesize that adjusting the abundance of specific intestinal bacteria through antibiotics would influence the occurrence and progression of ICI-associated myocarditis. This hypothesis has now been confirmed by numerous preclinical studies. Existing studies have demonstrated that the abundance of specific gut bacteria, such as *Flexispira* and *Ruminococcaceae*, showed a positive correlation with cardiac injury markers. Reducing their levels with antibiotics attenuated inflammatory cell infiltration and diminished myocardial injury area. An increasing number of specific intestinal bacteria linked to ICI-associated myocarditis are expected to be discovered. Targeting these specific intestinal bacteria with antibiotics may emerge as a new therapeutic strategy for ICI-associated myocarditis. At the same time, the use of antibiotics to mitigate irAEs may also compromise the efficacy of ICIs therapy. Selecting the right type and dosage of antibiotics to mitigate ICI-associated myocarditis without compromising the efficacy of ICIs therapy represents a key focus for future research.

Meanwhile, prior research demonstrated that incidence of ICI-related cardiac adverse events and common cardiotoxic manifestations varied within cancer types (Dong et al., 2022). The incidence of ICI-associated myocarditis mediated by the gut microbiota is also likely heterogeneous across different cancer types. These differences warrant further investigation in the future.

Current research on the relationship between gut microbiota and ICI-associated myocarditis remains in its infancy. Prior evidence has established the rationale for modulating the gut microbiota to improve the incidence and prognosis of ICI-associated myocarditis. In the future, we may prevent or treat ICI-associated myocarditis by regulating the gut microbiota through methods such as microbiota transplantation, antibiotic regimens, or probiotic supplements (Spencer et al., 2021; Lu et al., 2022). For cancer patients receiving ICI treatment, this holds significant promise.
